# 2-Amino-3-carb­oxy­pyrazin-1-ium nitrate monohydrate

**DOI:** 10.1107/S1600536811003126

**Published:** 2011-01-29

**Authors:** Fadila Berrah, Amira Ouakkaf, Sofiane Bouacida, Thierry Roisnel

**Affiliations:** a Laboratoire de Chimie Appliquée et Technologie des Matériaux, Université Larbi Ben M’Hidi, 04000 Oum El Bouaghi, Algeria; b Département Sciences de la Matière, Faculté des Sciences Exactes et Sciences de la Nature et de la Vie, Université Larbi Ben M’hidi, 04000 Oum El Bouaghi, Algeria; c Unité de Recherche de Chimie de l’Environnement et Moléculaire Structurale, CHEMS, Faculté des Sciences Exactes, Université Mentouri Constantine 25000, Algeria; d Centre de Difractométrie X, UMR 6226 CNRS Unité Sciences Chimiques de Rennes, Université de Rennes I, 263 Avenue du général Leclerc, 35042 Rennes, France

## Abstract

In crystal structure of the title compound, C_5_H_6_N_3_O_2_^+^·NO_3_^−^·H_2_O, inter­molecular N—H⋯O, O—H⋯N and O—H⋯O hydrogen bonds link the cations, anions and water mol­ecules into ribbons extending in [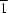
10]. Weak inter­molecular C—H⋯O hydrogen bonds further link these ribbons into sheets parallel to (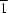 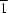
3).

## Related literature

For similar compounds, see: Berrah *et al.* (2005*a* ▶,*b* ▶); Bouacida *et al.* (2005, ▶ 2009 ▶); Dobson & Gerkin (1996 ▶). For hydrogen-bond graph-set motifs, see: Etter *et al.* (1990 ▶); Bernstein *et al.* (1995 ▶).
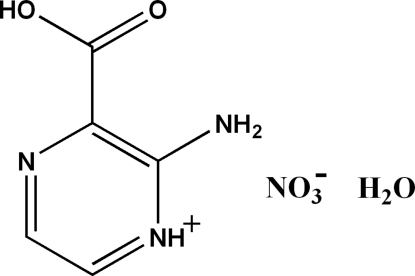


## Experimental

### 

#### Crystal data

C_5_H_6_N_3_O_2_^+^·NO_3_^−^·H_2_O
*M* *_r_* = 220.15
Triclinic, 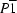

*a* = 5.1277 (4) Å
*b* = 7.6368 (6) Å
*c* = 12.1571 (10) Å
α = 97.872 (3)°
β = 100.588 (3)°
γ = 106.194 (3)°
*V* = 440.37 (6) Å^3^
*Z* = 2
Mo *K*α radiation
μ = 0.15 mm^−1^
*T* = 150 K
0.58 × 0.49 × 0.42 mm

#### Data collection

Bruker APEXII diffractometer
Absorption correction: multi-scan (*SADABS*; Sheldrick, 2002 ▶) *T*_min_ = 0.773, *T*_max_ = 0.938
5333 measured reflections
1967 independent reflections
1693 reflections with *I* > 2σ(*I*)
*R*_int_ = 0.028

#### Refinement

*R*[*F* ^2^ > 2σ(*F* ^2^)] = 0.036
*wR*(*F* ^2^) = 0.097
*S* = 1.03
1967 reflections
143 parameters
H atoms treated by a mixture of independent and constrained refinement
Δρ_max_ = 0.36 e Å^−3^
Δρ_min_ = −0.25 e Å^−3^

### 

Data collection: *APEX2* (Bruker, 2001 ▶); cell refinement: *SAINT* (Bruker, 2001 ▶); data reduction: *SAINT*; program(s) used to solve structure: *SIR2002* (Burla *et al.*, 2003 ▶); program(s) used to refine structure: *SHELXL97* (Sheldrick,2008 ▶); molecular graphics: *ORTEP-3 for Windows* (Farrugia, 1997 ▶) and *DIAMOND* (Brandenburg & Berndt, 2001 ▶); software used to prepare material for publication: *WinGX* (Farrugia, 1999 ▶).

## Supplementary Material

Crystal structure: contains datablocks global, I. DOI: 10.1107/S1600536811003126/cv5043sup1.cif

Structure factors: contains datablocks I. DOI: 10.1107/S1600536811003126/cv5043Isup2.hkl

Additional supplementary materials:  crystallographic information; 3D view; checkCIF report

## Figures and Tables

**Table 1:** Hydrogen-bond geometry (Å, °)

*D*—H⋯*A*	*D*—H	H⋯*A*	*D*⋯*A*	*D*—H⋯*A*
O1—H1⋯O1*W*^i^	0.84	1.69	2.5233 (17)	168
O1*W*—H1*W*⋯O5^ii^	0.80 (3)	1.93 (3)	2.7152 (18)	167 (3)
O1*W*—H2*W*⋯O1	0.88 (3)	2.39 (3)	2.8825 (19)	116 (2)
O1*W*—H2*W*⋯N4	0.88 (3)	1.99 (3)	2.8566 (18)	170 (2)
N2—H2*A*⋯O5	0.88	2.01	2.8549 (17)	161
N2—H2*B*⋯O2	0.88	2.08	2.7163 (17)	128
N2—H2*B*⋯O2^iii^	0.88	2.20	2.9125 (18)	137
N3—H3⋯O4	0.88	1.91	2.7825 (16)	174
C4—H4*A*⋯O4^iv^	0.95	2.24	3.1818 (17)	169

Symmetry codes: (i) 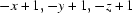; (ii) 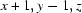; (iii) 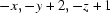; (iv) 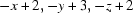.

**Fig. 1.**
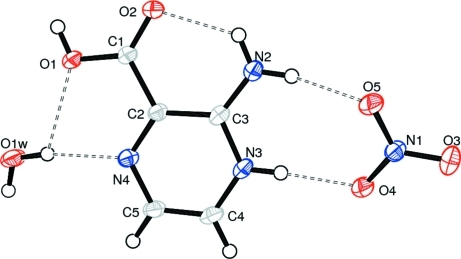
Ortep-3 (Farrugia, 1997) view of (I) showing the atomic labelling scheme. Displacement ellipsoids are drawn at the 50% probability level. Hydrogen bonds are shown as dashed lines.

**Fig. 2.**
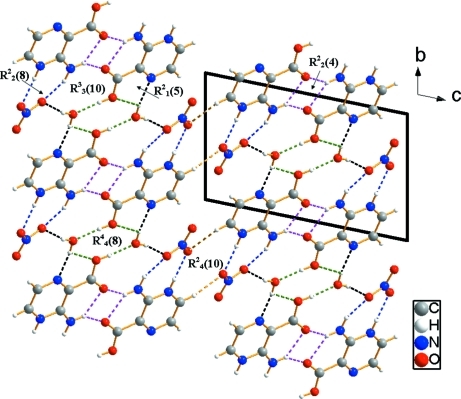
DIAMOND (Brandenburg & Berndt, 2001) view of a portion of hydrogen-bonded sheet in (I) showing the graph set motif notations. Hydrogen bonds are shown as dashed lines.

## supplementary crystallographic information

### Comment

As a part of our search for new hybrid compounds based on protonated amines (Berrah *et al.* 2005**a*,b*; Bouacida *et al.* 2005; 2009), we present the crystal structure of the title compound, (I).

The asymmetric unit of (I) contains one cation, one anion and one water molecule linked trough hydrogen bonds (Fig. 1). Bond distances and angles are similar to those encountered in analogous compounds (Berrah *el al.* 2005*a,b*; Dobson & Gerkin, 1996).

The crystal packing in the title structure can be described by considering sheets parallel to (-1-13) plane (Fig. 2). A sheet is an alternation of ribbons joined by a weak hyrogen bonds C4—H4···O4 and extended in direction [-110] (Fig. 2, Table 1). 3-Amino-pyrazinium 2-carboxylic acid cations, of the same ribbon, form centrosymetric dimers *via* N2—H2B···O2 hyrogen bonds. Each dimer is surrounded by two NO_3_^-^ anions and four H_2_O molecules, and all its atoms (except C5) are involved in N—H···O, O—H···N and O—H···O H-bonds. While nitrate anions are only acceptor of H-bonds, water molecules are at the same time donor and acceptor (Table 1). The resulting 2D hydrogen-bonded network exhibit rings with *R*_4_^4^(8), *R*_2_^4^(10), *R*_2_^2^(8), *R*_3_^3^(10), *R*_2_^2^(4) and *R*_2_^1^(5) graph set motifs (Etter *et al.*, 1990; Bernstein *et al.*, 1995) (Fig. 2).

### Experimental

The title compound was synthesized by reacting 3-amino-pyrazine 2- carboxylic acid with some excess of nitric acid in aqueous solution. Slow evaporation leads to well crystallized yellow needles.

### Refinement

H atoms of water molecule were located in difference Fourier map and included in the subsequent refinement with *U*_iso_(H) = 1.5*U*_eq_(O). The remaining H atoms were localized on Fourier maps but introduced in calculated positions and treated as riding on their parent atoms, with C—H = 0.95 Å, O—H = 0.84 Å and N—H = 0.88 Å, and with *U*_iso_(H) = 1.2 *U*_eq_(C or N) and *U*_iso_(H = 1.5 *U*_eq_(O).

### Crystal data

**Table:**

C_5_H_6_N_3_O_2_^+^·NO_3_^−^·H_2_O	*Z* = 2
*M**_r_* = 220.15	*F*(000) = 228
Triclinic, *P*1	*D*_x_ = 1.66 Mg m^−^^3^
*a* = 5.1277 (4) Å	Mo *K*α radiation, λ = 0.71073 Å
*b* = 7.6368 (6) Å	Cell parameters from 2815 reflections
*c* = 12.1571 (10) Å	θ = 2.8–27.5°
α = 97.872 (3)°	µ = 0.15 mm^−^^1^
β = 100.588 (3)°	*T* = 150 K
γ = 106.194 (3)°	Prism, yellow
*V* = 440.37 (6) Å^3^	0.58 × 0.49 × 0.42 mm

### Data collection

**Table:**

Bruker APEXII diffractometer	1693 reflections with *I* > 2σ(*I*)
graphite	*R*_int_ = 0.028
CCD rotation images, thin slices scans	θ_max_ = 27.5°, θ_min_ = 2.8°
Absorption correction: multi-scan (*SADABS*; Sheldrick, 2002)	*h* = −6→6
*T*_min_ = 0.773, *T*_max_ = 0.938	*k* = −9→9
5333 measured reflections	*l* = −15→15
1967 independent reflections	

### Refinement

**Table:**

Refinement on *F*^2^	Primary atom site location: structure-invariant direct methods
Least-squares matrix: full	Secondary atom site location: difference Fourier map
*R*[*F*^2^ > 2σ(*F*^2^)] = 0.036	Hydrogen site location: inferred from neighbouring sites
*wR*(*F*^2^) = 0.097	H atoms treated by a mixture of independent and constrained refinement
*S* = 1.03	*w* = 1/[σ^2^(*F*_o_^2^) + (0.0451*P*)^2^ + 0.1681*P*] where *P* = (*F*_o_^2^ + 2*F*_c_^2^)/3
1967 reflections	(Δ/σ)_max_ < 0.001
143 parameters	Δρ_max_ = 0.36 e Å^−^^3^
0 restraints	Δρ_min_ = −0.25 e Å^−^^3^

### Special details

**Table:**

Geometry. All e.s.d.'s (except the e.s.d. in the dihedral angle between two l.s. planes) are estimated using the full covariance matrix. The cell e.s.d.'s are taken into account individually in the estimation of e.s.d.'s in distances, angles and torsion angles; correlations between e.s.d.'s in cell parameters are only used when they are defined by crystal symmetry. An approximate (isotropic) treatment of cell e.s.d.'s is used for estimating e.s.d.'s involving l.s. planes.
Refinement. Refinement of *F*^2^ against ALL reflections. The weighted *R*-factor *wR* and goodness of fit *S* are based on *F*^2^, conventional *R*-factors *R* are based on *F*, with *F* set to zero for negative *F*^2^. The threshold expression of *F*^2^ > σ(*F*^2^) is used only for calculating *R*-factors(gt) *etc*. and is not relevant to the choice of reflections for refinement. *R*-factors based on *F*^2^ are statistically about twice as large as those based on *F*, and *R*- factors based on ALL data will be even larger.

### Fractional atomic coordinates and isotropic or equivalent isotropic displacement parameters (Å^2^)

**Table:**

	*x*	*y*	*z*	*U*_iso_*/*U*_eq_	
C1	0.3536 (3)	0.81463 (18)	0.56444 (11)	0.0213 (3)	
C2	0.5373 (3)	0.93343 (17)	0.67594 (11)	0.0196 (3)	
C3	0.5053 (3)	1.11068 (18)	0.71720 (11)	0.0206 (3)	
C4	0.8826 (3)	1.14836 (19)	0.87398 (11)	0.0243 (3)	
H4A	1.0058	1.2218	0.9434	0.029*	
C5	0.9034 (3)	0.97938 (19)	0.83049 (11)	0.0239 (3)	
H5	1.0420	0.9354	0.8702	0.029*	
N1	0.4633 (3)	1.60420 (16)	0.88650 (10)	0.0226 (3)	
N2	0.3198 (3)	1.18108 (16)	0.66687 (11)	0.0270 (3)	
H2A	0.3137	1.2905	0.6980	0.032*	
H2B	0.2019	1.1188	0.6021	0.032*	
N3	0.6850 (2)	1.20958 (15)	0.81712 (10)	0.0228 (3)	
H3	0.6719	1.3181	0.8460	0.027*	
N4	0.7294 (2)	0.87393 (15)	0.73168 (9)	0.0219 (3)	
O1	0.3992 (2)	0.65591 (14)	0.54062 (9)	0.0303 (3)	
H1	0.3004	0.5977	0.4759	0.045*	
O2	0.1873 (2)	0.86840 (14)	0.50431 (9)	0.0288 (3)	
O3	0.4414 (2)	1.75010 (14)	0.93506 (9)	0.0326 (3)	
O4	0.6850 (2)	1.56230 (14)	0.91237 (9)	0.0279 (3)	
O5	0.2640 (2)	1.49165 (15)	0.80957 (9)	0.0337 (3)	
O1W	0.8473 (3)	0.53972 (18)	0.65527 (11)	0.0490 (4)	
H1W	0.979 (6)	0.543 (4)	0.704 (3)	0.074*	
H2W	0.793 (6)	0.637 (4)	0.671 (2)	0.074*	

### Atomic displacement parameters (Å^2^)

**Table:**

	*U*^11^	*U*^22^	*U*^33^	*U*^12^	*U*^13^	*U*^23^
C1	0.0231 (6)	0.0211 (6)	0.0170 (6)	0.0071 (5)	0.0009 (5)	0.0001 (5)
C2	0.0215 (6)	0.0196 (6)	0.0156 (6)	0.0051 (5)	0.0021 (5)	0.0017 (5)
C3	0.0209 (6)	0.0205 (6)	0.0174 (6)	0.0035 (5)	0.0043 (5)	−0.0001 (5)
C4	0.0246 (7)	0.0254 (7)	0.0157 (6)	0.0011 (5)	0.0002 (5)	0.0009 (5)
C5	0.0236 (7)	0.0255 (7)	0.0178 (6)	0.0050 (5)	−0.0014 (5)	0.0029 (5)
N1	0.0265 (6)	0.0208 (5)	0.0185 (6)	0.0066 (5)	0.0036 (5)	0.0014 (4)
N2	0.0284 (6)	0.0234 (6)	0.0255 (6)	0.0119 (5)	−0.0023 (5)	−0.0038 (5)
N3	0.0253 (6)	0.0195 (5)	0.0189 (6)	0.0049 (5)	0.0017 (5)	−0.0029 (4)
N4	0.0242 (6)	0.0212 (5)	0.0173 (6)	0.0055 (5)	0.0011 (4)	0.0023 (4)
O1	0.0380 (6)	0.0252 (5)	0.0214 (5)	0.0159 (4)	−0.0088 (4)	−0.0075 (4)
O2	0.0300 (5)	0.0276 (5)	0.0243 (5)	0.0135 (4)	−0.0071 (4)	−0.0016 (4)
O3	0.0406 (6)	0.0231 (5)	0.0337 (6)	0.0116 (5)	0.0108 (5)	−0.0023 (4)
O4	0.0256 (5)	0.0275 (5)	0.0250 (5)	0.0093 (4)	−0.0031 (4)	−0.0030 (4)
O1W	0.0619 (9)	0.0472 (7)	0.0308 (6)	0.0396 (7)	−0.0220 (6)	−0.0188 (5)
O5	0.0284 (6)	0.0316 (6)	0.0316 (6)	0.0109 (4)	−0.0085 (4)	−0.0070 (4)

### Geometric parameters (Å, °)

**Table:**

C1—O2	1.2162 (17)	C5—H5	0.9500
C1—O1	1.3017 (16)	N1—O3	1.2314 (14)
C1—C2	1.5050 (18)	N1—O4	1.2621 (15)
C2—N4	1.3132 (17)	N1—O5	1.2635 (15)
C2—C3	1.4420 (17)	N2—H2A	0.8800
C3—N2	1.3150 (18)	N2—H2B	0.8800
C3—N3	1.3580 (17)	N3—H3	0.8800
C4—N3	1.3488 (18)	O1—H1	0.8400
C4—C5	1.3663 (19)	O1W—H1W	0.81 (3)
C4—H4A	0.9500	O1W—H2W	0.88 (3)
C5—N4	1.3520 (17)		
			
O2—C1—O1	125.51 (12)	C4—C5—H5	119.6
O2—C1—C2	121.65 (11)	O3—N1—O4	121.00 (12)
O1—C1—C2	112.82 (12)	O3—N1—O5	120.97 (12)
N4—C2—C3	121.68 (12)	O4—N1—O5	118.02 (11)
N4—C2—C1	118.46 (11)	C3—N2—H2A	120.0
C3—C2—C1	119.83 (12)	C3—N2—H2B	120.0
N2—C3—N3	118.84 (12)	H2A—N2—H2B	120.0
N2—C3—C2	125.70 (12)	C4—N3—C3	122.72 (11)
N3—C3—C2	115.46 (12)	C4—N3—H3	118.6
N3—C4—C5	119.16 (12)	C3—N3—H3	118.6
N3—C4—H4A	120.4	C2—N4—C5	120.09 (11)
C5—C4—H4A	120.4	C1—O1—H1	109.5
N4—C5—C4	120.89 (13)	H1W—O1W—H2W	110 (3)
N4—C5—H5	119.6		
			
O2—C1—C2—N4	−174.02 (13)	N3—C4—C5—N4	0.1 (2)
O1—C1—C2—N4	4.43 (18)	C5—C4—N3—C3	−0.5 (2)
O2—C1—C2—C3	4.1 (2)	N2—C3—N3—C4	−179.55 (13)
O1—C1—C2—C3	−177.47 (12)	C2—C3—N3—C4	0.68 (19)
N4—C2—C3—N2	179.80 (13)	C3—C2—N4—C5	0.1 (2)
C1—C2—C3—N2	1.8 (2)	C1—C2—N4—C5	178.13 (12)
N4—C2—C3—N3	−0.45 (19)	C4—C5—N4—C2	0.1 (2)
C1—C2—C3—N3	−178.48 (11)		

### Hydrogen-bond geometry (Å, °)

**Table:**

*D*—H···*A*	*D*—H	H···*A*	*D*···*A*	*D*—H···*A*
O1—H1···O1W^i^	0.84	1.69	2.5233 (17)	168
O1W—H1W···O5^ii^	0.80 (3)	1.93 (3)	2.7152 (18)	167 (3)
O1W—H2W···O1	0.88 (3)	2.39 (3)	2.8825 (19)	116 (2)
O1W—H2W···N4	0.88 (3)	1.99 (3)	2.8566 (18)	170 (2)
N2—H2A···O5	0.88	2.01	2.8549 (17)	161
N2—H2B···O2	0.88	2.08	2.7163 (17)	128
N2—H2B···O2^iii^	0.88	2.20	2.9125 (18)	137
N3—H3···O4	0.88	1.91	2.7825 (16)	174
C4—H4A···O4^iv^	0.95	2.24	3.1818 (17)	169

Symmetry codes: (i) −*x*+1, −*y*+1, −*z*+1; (ii) *x*+1, *y*−1, *z*; (iii) −*x*, −*y*+2, −*z*+1; (iv) −*x*+2, −*y*+3, −*z*+2.
